# Intensive diagnostic management of coronavirus disease 2019 (COVID-19) in academic settings in Japan: challenge and future

**DOI:** 10.1186/s41232-020-00147-2

**Published:** 2020-10-12

**Authors:** Tokio Hoshina, Hiroka Aonuma, Manabu Ote, Tatsuya Sakurai, Erisha Saiki, Yuki Kinjo, Kazuhiro Kondo, Masataka Okabe, Hirotaka Kanuka

**Affiliations:** 1 Team COVID-19 PCR Center, The Jikei University School of Medicine, 3-25-8, Nishi-Shinbashi, Minato-ku, Tokyo, 105-8461 Japan; 2 Department of Infectious Diseases and Infection Control, The Jikei University School of Medicine, 3-25-8, Nishi-Shinbashi, Minato-ku, Tokyo, 105-8461 Japan; 3 Department of Tropical Medicine, The Jikei University School of Medicine, 3-25-8, Nishi-Shinbashi, Minato-ku, Tokyo, 105-8461 Japan; 4 Laboratory Animal Facilities, The Jikei University School of Medicine, 3-25-8, Nishi-Shinbashi, Minato-ku, Tokyo, 105-8461 Japan; 5 Department of Bacteriology, The Jikei University School of Medicine, 3-25-8, Nishi-Shinbashi, Minato-ku, Tokyo, 105-8461 Japan; 6 Department of Virology, The Jikei University School of Medicine, 3-25-8, Nishi-Shinbashi, Minato-ku, Tokyo, 105-8461 Japan; 7 Department of Anatomy, The Jikei University School of Medicine, 3-25-8, Nishi-Shinbashi, Minato-ku, Tokyo, 105-8461 Japan

**Keywords:** COVID-19, SARS-CoV-2, Virus, Diagnosis, PCR

## Abstract

Coronavirus disease 2019 (COVID-19), caused by severe acute respiratory syndrome coronavirus 2 (SARS-CoV-2), first emerged in Wuhan, China, and has spread globally to most countries. In Japan, the first COVID-19 patient was identified on January 15, 2020. By June 30, the total number of patients diagnosed with COVID-19 reached 18,000. The impact of molecular detection of pathogens is significant in acute-care settings where rapid and accurate diagnostic measures are critical for decisions in patient treatment and outcomes of infectious diseases. Polymerase chain reaction (PCR)-based methods, such as quantitative PCR (qPCR), are the most established gene amplification tools and have a comprehensive range of clinical applications, including detecting a variety of pathogens, even novel agents causing emerging infections. Because SARS-CoV-2 contains a single-stranded RNA genome, reverse-transcription qPCR (RT-qPCR) has been broadly employed for rapid and sensitive quantitative measurements of viral RNA copy numbers. The RT-qPCR method, however, still requires time-consuming reactions with two different enzymes in addition to isolation of RNA from patient samples, limiting the numbers of testing institutions for diagnosing SARS-CoV-2 infection. Japan is known to have performed a relatively small number of PCR tests as well as confirmed cases among developed nations; as of June 30, 2020, approximately 390,000 people in Japan had undergone PCR tests. Given the devastating impact on medical services and the scale of demand for diagnostic testing of COVID-19, it has been proposed that academic settings such as basic research departments in university/college can be engaged in diagnosing, especially in university hospitals or academic medical centers. In collaboration with established diagnostic laboratories, academic facilities can divert their function to detecting virus from patients with suspected COVID-19, adopting existing specialized expertise in virus handling, molecular work, and data analysis. This in-house testing strategy facilitates the rapid diagnosing of thousands of samples per day and reduces sample turnaround time from 1 week to less than 24 h. This review provides an overview of the general principles, diagnostic value, and limitations of COVID-19 diagnosis platforms in Japan, in particular in-house testing at academic settings.

## Background

The emergence of the novel coronavirus, severe acute respiratory syndrome coronavirus 2 (SARS-CoV-2), has led to a pandemic of coronavirus disease (COVID-19), posing a major global public health crisis [1–3]. Timely and adequate treatment for COVID-19 requires the rapid and accurate detection of SARS-CoV-2 in the acute-care setting. The previous 2003 SARS epidemic (severe acute respiratory syndrome coronavirus (SARS-CoV)) and 2009 H1N1 influenza pandemic (H1N1pdm09 virus) also indicated the importance of rapid diagnostics for decision-making with regard to medical triage, infection control, and patient treatment [4]. Regardless of the fact that the outcomes from infection rely on how quickly the pathogen is detected, however, hospital laboratories still remain conventional in their use of traditional, time-consuming, multistep methods such as culture-based assays to diagnose emerging and re-emerging diseases, which occasionally fail to satisfy the demand of the diagnosis in the acute and critical-care settings [5, 6].

PCR-based methods are the most promising molecular diagnostic tools for infectious diseases in medical settings [7]. Reverse-transcription quantitative PCR (RT-qPCR) is a highly reliable and mature method that has been used worldwide for the diagnosis of COVID-19 via detecting a viral RNA from the nasopharynx, saliva, or other tissues of suspected patients [8]. RT-qPCR with specific primers identifies the presence of RNA sequences of the SARS-CoV-2 gene encoding proteins such as the nucleocapsid of the virus. Each reaction amplifying the viral gene sequence causes the cleavage of a fluorescent probe, inducing a signal that increases in intensity with each cycle. The fluorescence intensity indicates what quantity of viral RNA is present in the patient sample [9].

With increasing numbers of sequenced genomes of emerging and re-emerging pathogens, a variety of gene databases has been consolidated to select candidate amplification targets applicable for designing primers of the PCR tests. Numerous variations of commercial PCR assays for pathogen detection have been developed in recent decades, and public institutions and private testing laboratories continue to install these assays. Whereas detecting and analyzing PCR products have typically been automated, the overall processes are still laborious and time-consuming, which often diminishes the clinical applicability of molecular diagnosis [10]. In the COVID-19 pandemic, inevitable delays in rapid diagnostic testing for SARS-CoV-2 infection have happened in many countries even in hospital-based laboratories equipped with RT-qPCR assay systems. It is apparent that rapid diagnosis is better able to lead to appropriate treatment for patients with COVID-19 and better public health management [11].

This review examines the potential value of academic settings as reliable in-house testing infrastructure satisfying the urgent need for rapid diagnosis. This review introduces the development of an academic platform of SARS-CoV-2 diagnostic RT-qPCR assay with rapid turnaround time, citing a practical example in Japan, and provides the pros and cons, and new insights on molecular diagnosis in the pandemic era.

## Diagnosis of COVID-19

### Clinical diagnosis

COVID-19 is clinically diagnosed based on disease manifestation and the patient’s characteristics such as epidemiological background, interview of travel history, sickness among contacts, and laboratory findings [12–14]. COVID-19 infection may cause one or several symptoms including cough, shortness of breath, fever, chills, muscle pain, headache, sore throat, and loss of taste and/or smell [12, 13, 15]. The incubation period is generally 2–14 days after exposure to SARS-CoV-2 [16]. The symptoms in the early phase sometimes resemble those of influenza and the common cold; thus, clinicians often face difficulties in distinguishing COVID-19 from other infections. Moreover, subclinical and asymptomatic SARS-CoV-2 infection is quite common. These features make adequate diagnosis extremely difficult.

Occasionally, COVID-19 patients develop pneumonia, which can progress to severe respiratory failure and death [17]. Typical manifestation in chest computed tomography (CT) imaging provides a fair clinical diagnosis of COVID-19 [18, 19]. A retrospective cohort study (99 chest CT images including 51 COVID-19 patients and two patients of adenovirus) demonstrated that 90.2% of cases showed ground-glass opacity (GGO) in chest CT imaging [20]. However, the distribution of the GGO area is non-specific and does not directly indicate infection with SARS-CoV-2; the misidentification rate was 3.9% in the same study [20]. The large number of COVID-19 patients showing asymptomatic and mild symptoms suggests that CT imaging alone should not be used for definitive diagnosis of COVID-19 [21]. In order to achieve definitive diagnosis, laboratory testing confirmation, such as RT-qPCR for detecting the virus from saliva of the oral cavity and lower or upper respiratory samples is essential in addition to chest CT scanning.

### PCR-based method

RT-qPCR has been and is still deemed as the gold standard to diagnose COVID-19. This method based on PCR has high accuracy and high sensitivity with a detection limit of less than 10 RNA copies [22] and permits medium-throughput testing using large format plates. RT-qPCR detects SARS-CoV-2 by amplifying the target cDNA sequence reverse-transcribed from the viral RNA using a reverse transcriptase such as M-MLV Reverse Transcriptase. Viral RNA isolation from specimens with various commercial kits has been reported, and moreover, RNA isolation steps may be eliminated by simple approaches such as heating or direct transfer in viral (universal) transport media [23–26]. One-step commercial master mixes omitting a separate reverse transcription step have also been tested and approved for the detection of SARS-CoV-2 [22, 27]. Besides, development of in-house protocols using kits or reagents for normal laboratory use provides more flexibility for diagnostic methods especially in pandemic situations. To detect SARS-CoV-2, one or multiple primer sets targeting the viral E (envelope), RdRP (RNA-dependent RNA polymerase), and N (nucleoprotein) genes have been implemented [28]. Both fluorescent probes and specific oligonucleotides annealing to viral cDNAs allow high specificity. For example, the probe is labeled with a fluorophore at the 5’-end and a quencher at 3’-end in our method. The fluorophore is released from the quencher with degradation of the probes by the exonuclease activity of DNA polymerase, thereby enabling detection of real-time amplification by measuring fluorescence intensity using a qPCR instrument.

### Isothermal amplification method

Loop-mediated isothermal amplification (LAMP) is a rapid and simple DNA amplification method, in which the reaction proceeds in isothermal conditions [29]. Many detection methods based on LAMP or reverse transcription (RT)-LAMP targeting infectious diseases, including viral, bacterial, and parasitic diseases, have been developed and shown their potential to be utilized for epidemiological analysis [30–34]. RT-LAMP has already been validated to detect RNA viruses, including influenza, SARS, and MERS [35–37]. After the emergence of COVID-19, several groups have developed and reported RT-LAMP methods to detect RNA of SARS-CoV-2 and yielded similar sensitivity compared with those utilizing RT-qPCR [38, 39]. Recently, a SARS-CoV-2 diagnostic kit based on RT-LAMP was also approved and released in Japan (Eiken Chemical Co. Ltd.).

LAMP often uses *Bst* polymerase, which synthesizes a new DNA strand with simultaneous displacement of former complementary strand. The LAMP reaction is achieved with four specific primers recognizing six regions of the target gene, providing high specificity. In this reaction, a stem-loop DNA structure is formed by contribution of two outer primers, and DNA synthesis continues with two inner primers, each consisting of the connected sequences of the same and complementary sequences to the inner two regions of the stem-loop structure. This reaction thus produces various sized structures in an amplified product. In RT-LAMP, reverse transcription proceeds simultaneously with DNA amplification, thereby allowing one-step detection. Prominent advantages of RT-LAMP are its easy handling and fast readout. All reactions can be achieved at a constant temperature; thus, only a heating block or hot water is necessary for testing. The result can be detected using a real-time turbidimeter or by the naked eye by observing the accumulation of magnesium pyrophosphate, a by-product of the reaction. Addition of a visualizing reagent, such as calcein and phenol red, enables easier visual detection [38–40]. The simplicity may advance RT-LAMP to a prospective point-of-care method to diagnose COVID-19.

### Antibody and antigen test

SARS-CoV-2 infection elicits protective immune response and promotes immunoglobulin M and G antibody production against virus antigens such as spike glycoprotein [41]. Previous clinical studies on SARS-CoV and MERS-CoV showed that virus-specific immunoglobulins were present in more than 80% of infected patients at least 4 weeks after the onset of symptom [42–45]. Serology testing to detect SARS-CoV-2-specific antibodies, including ELISA, virus neutralization assay, and lateral flow (dipstick) assay, were rapidly developed and are now available for COVID-19 diagnosis [46–50]. A test detecting SARS-CoV-2 immunoglobulin G demonstrated that virus-specific antibodies were detectable from 100% of patients within 19 days after onset of symptoms [51]. The serological test does not indicate the presence of SARS-CoV-2 itself, but does that of antibodies against the virus. The seroconversion of the virus-specific antibodies takes at least 5 days after onset of symptoms [52, 53]. It has also been pointed out that SARS-CoV-2 can be transmitted before the onset of symptoms or from asymptomatic patients [54], suggesting that infection would occur before the antibody titer was raised and antibody detection alone should not be used for diagnosis of COVID-19. Antibody detection helps to determine previous exposure to SARS-CoV-2, providing an estimated number of COVID-19 cases, which is essential for the management of infection control. The fact that immunity induced by initial infection protects against reinfection in rhesus macaques and hamster models indicates the possibility of effective vaccine development against SARS-CoV-2 [55–57]. In order to establish prevention against SARS-CoV-2 and stamp out the coronavirus pandemic, further and detailed examinations on antigen and antibodies related to COVID-19, such as how long the antibodies persist in infected patients, are needed.

## Diagnostic platforms for COVID-19 in Japan

Policies for COVID-19 testing vary between countries, resulting in significant differences in the numbers of tests performed in each country. As for Japan, a relatively small number of PCR tests has been conducted among developed nations; as of June 30, 2020, approximately 390,000 Japanese people had undergone PCR tests. The epidemic curve in Japan has been relatively gentle, and the number of confirmed death cases are small compared with those of other heavily affected countries. Here we discuss the COVID-19 testing strategy implemented in Japan: the emphasis is on features of the COVID-19 diagnostic platforms.

### National institution under government control

The National Institute of Infectious Diseases (NIID) is the central, national public health institute for research and development of infectious disease control and prevention in Japan. When a novel infectious disease such as COVID-19 emerges in Japan, it is initially reported to the NIID from hospitals through municipal public health institutes, or quarantine stations. The NIID identified the initial COVID-19 patient who had traveled to Wuhan and visited a hospital as the emergence of COVID-19 in Japan [58]. The NIID immediately isolated a causal virus and developed RT-qPCR protocols for the detection of the viral RNAs using specimens from patients. The NIID published the test protocols with guidance on sample collection and transportation on January 21, 2020. The protocols have been updated regularly referring to latest reports and subsequent examinations.

Following an increase in COVID-19 cases, a large-scale RT-qPCR test was implemented in municipal public health institutes and quarantine stations under the direction of the Ministry of Health, Labour and Welfare of Japan based on the NIID protocols, where the NIID distributed the reagents for RT-qPCR since January 30, 2020 [59]. The NIID has also performed the diagnostic testing of COVID-19 cases aboard the Diamond Princess cruise ship in February, 2020 [60], and also directed active epidemiological surveys to track COVID-19 in all prefectures in Japan [61]. Rather than acting as a diagnostic PCR center, the NIID had a role in establishing a standard diagnostic system feasible for public health institutes and quarantine stations.

### Public diagnostic facilities

Municipal public health institutes and quarantine stations are responsible for protecting public health and safety through the control and prevention of various diseases in Japan. They cover a wide range of inspections including infectious disease diagnostics, food-borne pathogen detection, and water quality surveys. A considerable number of public health institutes are already equipped with qPCR machines and automated nucleic acid extraction systems [62]. As countermeasures against outbreaks of seasonal influenza, municipal public health institutes and quarantine stations have been preparing for flu RT-qPCR tests in accordance with protocols developed by the NIID. The reagents including probes and primers were distributed from the NIID as well [63, 64]. These institutions have the ability to develop in-house protocols for RT-qPCR independently, which are based on nucleic acid sequences deposited on public databases, and launch virus tests in advance of the release of NIID protocols [65].

Municipal public health institutes and quarantine stations essentially lack enough human resources and equipment to control a state of emergency, because the aim of these institutions is to protect public health and safety in ordinary conditions. Even in an epidemic or pandemic, these institutions are still required to perform normal duties as usual. In the case of the 2009 swine flu pandemic, a tremendous number of PCR samples from patients overwhelmed the inspection capacity (although new equipment was quickly deployed at that time) [65]. In addition, it usually takes considerable time to transport patient samples from hospitals to local institutes or stations to perform PCR diagnostics, resulting in increasing turnaround times for testing.

### Commercial clinical laboratories

A clinical laboratory (or medical laboratory) is a commercial facility that provides clinical examination services to medical institutions to assist physicians’ clinical decisions. Their services cover a variety of specimens of both anatomical and clinical pathologies. For maintaining and assuring the quality of medical examinations provided, the laboratories are generally accredited and authorized by credentialing agencies. Each commercial clinical laboratory in Japan should be registered with the respective local government authority and also accredited by the Japan Accreditation Board (JAB) and the Japan Committee for Clinical Laboratory Standards (JCCLS) on the basis of international standard ISO15189 (Medical laboratories-requirements for quality and competence) [66]. The use of commercial clinical laboratories is cost-effective for reagent and labor costs, and they have been adopted widely in many countries. On the other hand, for the diagnosis of infectious diseases, the turnaround time of commercial clinical laboratories may be longer than that of in-house clinical laboratories, due to the fact that the transportation of infectious specimens to off-site laboratories is time-consuming [11].

COVID-19 has been officially designated as a “New Infectious Disease”, which is an infectious disease with specific case reporting requirements, as detailed in the law (“Prevention of Infectious Diseases and Medical Care for Patients with Infectious Diseases Act”) in Japan. Initially, official regulation allowed commercial clinical laboratories to accept COVID-19 patient samples only from the public health centers requesting diagnosis, for which expenses were not covered by insurance. To enhance testing capacity for COVID-19 in clinical laboratories, on March 6, 2020, the Ministry of Health, Labour and Welfare of Japan enabled medical institutions to request PCR testing directly from clinical laboratories, which is fully covered by public health insurance [67]. Commercial laboratories are expanding their capacities for COVID-19 diagnostics by implementing other measures such as SARS-CoV-2 antigen/antibody detection.

### Medical institutions

Protecting the health and safety of both patients and medical staff is one of the top priorities of each medical institution. Suspected patients with COVID-19 and close contacts (someone who has contacted a patient for 15 min or more without taking the necessary infection-prevention measures, at a distance within 1 m) have been generally directed to testing sites when healthcare professionals decide that these people need to be examined [68]. In order to prevent hospital infection and to secure high reliability of the test, it is required to collect samples in the proper manner at designated sites where a well-organized infection control program is implemented. In Japan, one of the designated sites for COVID-19 testing is a hospital that has officially been appointed as an outpatient facility for Japanese returnees and potential contacts. Information about these hospitals such as location or name are undisclosed, avoiding being flooded with patients. Patients with noticeable symptoms at first need to consult the Call Centers for Japanese Returnees and Potential Contacts or to visit advanced medical institutions directly. If the patient is deemed to be suspected as having COVID-19, the physician who examines those patients can order the test to a commercial clinical laboratory, a local public health institute, or a medical institution with PCR test capacity (effective on March 6, 2020) [69].

## COVID-19 diagnosis in academic settings in Japan

### Situation and ramifications of COVID-19 in Japan

In Japan, the numbers of infected and fatal cases of COVID-19 exceeded 18,000 and 900, respectively, in June 2020 [70]. In April 2020, confirmed cases of COVID-19 increased exponentially, and the healthcare systems in Japan were nearly overwhelmed by the burden [71]. A number of nosocomial COVID-19 infections occurred in hospitals including special functioning and regional medical care support hospitals, which resulted in suspension of their function [72].

COVID-19 was specified as a designated infectious disease in the Japanese Infectious Disease Control Law on January 28, 2020 [73]. The regulation forced not only patients positive for SARS-COV-2 infection but even asymptomatic positive persons to be hospitalized, in order to prevent the collapse of medical services. Hospitalization of COVID-19 patients in Tokyo was initially limited to only designated hospitals such as designated medical institutions for specified infectious diseases in addition to Tokyo metropolitan hospitals belonging to Tokyo Metropolitan Health and Hospitals Corporation [73]. One of the issues to be solved was how to increase the number of available hospital beds for patients, together with managing nosocomial infections.

### COVID-19 testing at academic laboratories: background

**Fig. 1:**
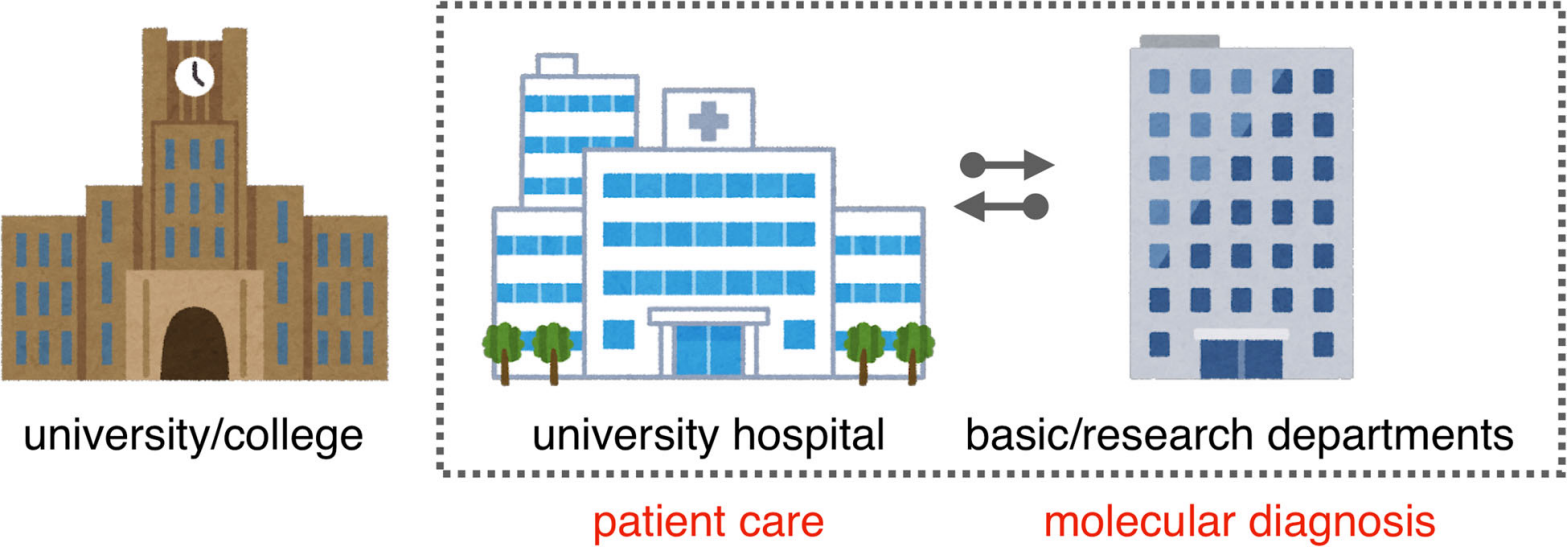
Schematic illustration of an in-house SARS-CoV-2 RT-PCR-based diagnostic assay in an academic setting, resulting in greatly improved turnaround times. Basic/research departments and institutes of a university, at which their hospital accepts patients in need of care for COVID-19, can be enabled to perform molecular diagnosis using commercial and laboratory-developed tests using research use-only reagents

Screening for SARS-CoV-2 using a PCR test on new admission has been preferred to avoid nosocomial infection when a high prevalence of COVID-19 patients are present in the community [74]. Testing pre-admission and pre-procedure is also recommended [75]. Patients with older age, hypertension, chronic heart failure, or neoplasms are at greater risk of developing severe COVID-19 symptoms [13]. In the meantime, in Japan, PCR testing was not allowed for either mild cases not developing pneumonitic reactions or most of the cases of patients who had already been admitted, mainly due to the limited capacity of PCR testing by public authorities. For managing infection control with limited resources, it appears to be an idea worth considering to introduce SARS-CoV-2 PCR testing to other facilities such as non-medical ones. Candidates for the implementation of SARS-CoV-2 PCR testing include academic settings, in particular, laboratories at universities with hospitals, to detect infections in both community and nosocomial settings. PCR testing in the same organization allows for both greater flexibility and rapidity to measure and act on infected cases (Fig. 1).

Japan has over 80 universities with their own hospitals, which are national, public, or private [76]. Each prefecture in Japan has at least one medical school, serving a function as a central medical institution in each area (most of them are referred to as Special Functioning Hospitals). A number of the university hospitals are located around large cities such as Tokyo, Osaka, Kyoto, Nagoya, Sapporo, Fukuoka, and Sendai, often being associated with branch hospitals located in suburban areas. Most of the 25 academic hospitals in the Tokyo area have accommodated COVID-19 patients during the pandemic, in accordance with accumulating experience of the management of COVID-19 cases. The establishment of SARS-CoV-2 testing systems in each academic institution is expected as a promising measure to increase testing capacity and obtain results quickly for prevention of nosocomial infections and maintaining patient treatment during the COVID-19 crisis. Although Japan’s relatively low rate of SARS-CoV-2 testing has been indicated, implementation of SARS-CoV-2 diagnostic pipeline in academic laboratories may be one of the solutions for the pandemic situation in many countries.

### COVID-19 testing at academic laboratories: a case

A private medical university, The Jikei University School of Medicine, launched an academic platform for detecting SARS-CoV-2 on February 14, 2020, almost a month before WHO declared the pandemic. COVID-19 cases in Japan had been increasing gradually after the spring festival in China [77]. At that time, SARS-CoV-2 PCR testing in the Tokyo area was performed only at the Tokyo Metropolitan Institute of Public Health. Any patients without pneumonia were not allowed to have a PCR examination, even if they showed other COVID-19-like symptoms such as fever, fatigue, and dry cough.

**Fig. 2:**
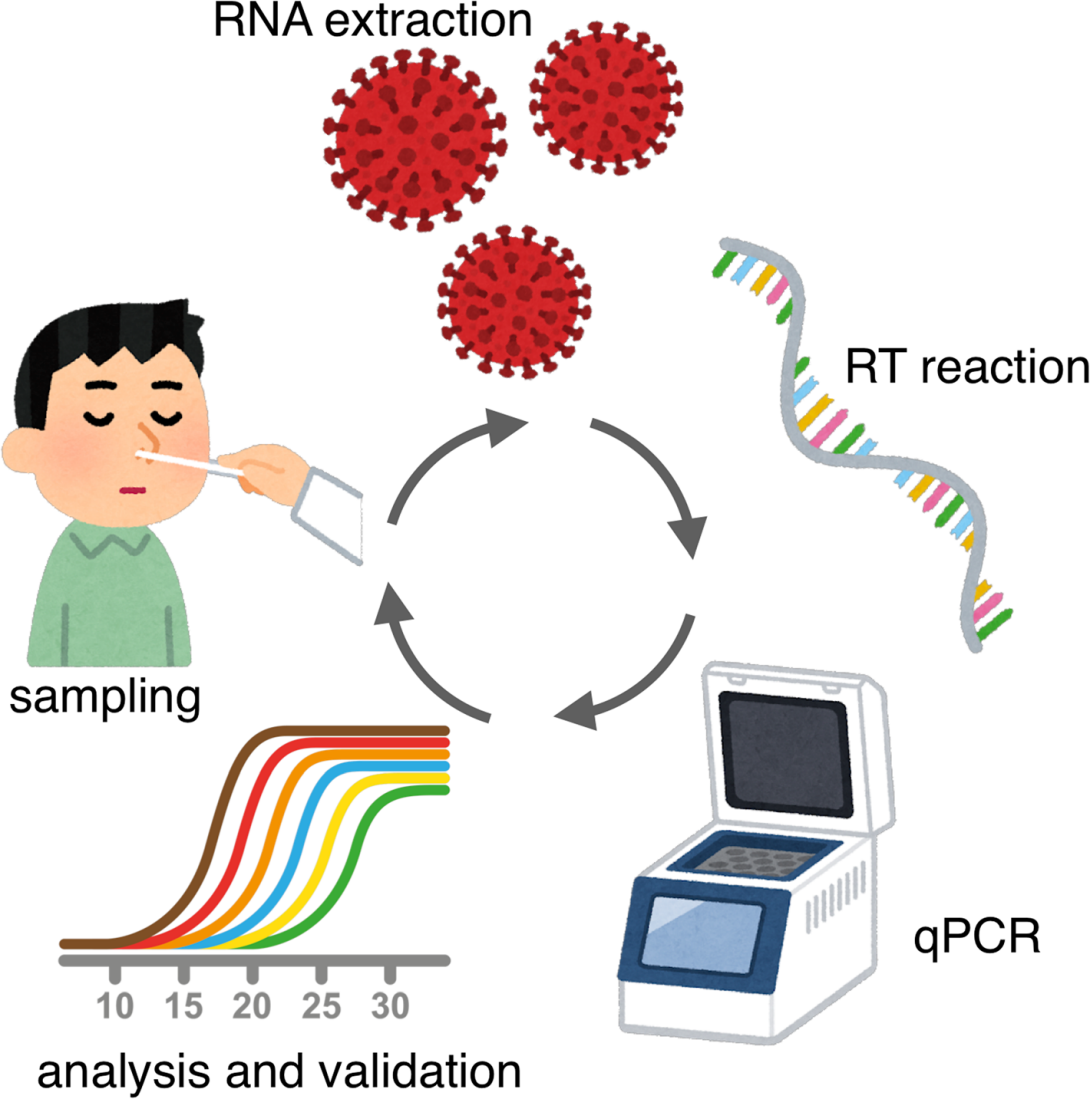
Schematic of workflow of an in-house RT-qPCR-based assay for detecting SARS-CoV-2, showing each step from patient sampling to reporting of results. A sample is collected from the patient’s nasopharynx, saliva, or other tissues. RNA is extracted from the sample and then transcribed into cDNA (RT reaction). DNA polymerase amplifies the cDNA, degrading fluorescent probes which results in an increased fluorescence intensity (qPCR). If the intensity reaches a certain threshold, the sample is classed as positive

The Jikei University School of Medicine with an affiliated hospital is located in the Minato district in central Tokyo. The university hospital is one of the largest hospitals in the district, supporting the local public health center. One of the major aims of installing this platform, named the Team COVID-19 PCR center, was to support the hospital laboratory in the same place. The PCR center was established on the basis of a cross-departmental collaboration; members from basic science departments, such as virology, bacteriology, and parasitology, participated in and operated the center. The members were highly skilled in handling both genes and pathogens, making the center competent to perform the detection of a pathogenic virus safely and reliably. The center used facilities of each department as a resource for virus detection, such as Biosafety Level 2 (BSL-2) rooms, equipment for RNA extraction, and qPCR machines, which were already installed at the departments. The SARS-CoV-2 RT-qPCR protocols were introduced by referring to the NIID protocol [78] and a previous report [79], with modifications to maximize its sensitivity and accuracy. It is important to note that the center was operated in collaboration between clinicians and researchers, which ensures sustainable management of the testing platform. For example, clinical information on patients and the results of tests were immediately shared between the university hospital and the center (Fig. 2).

The center has tested over 1900 clinical samples including ones not only from outpatients with possible infection but also from screening at hospital admission since the launch (as of July 12, 2020), while experiencing a nosocomial outbreak at the Jikei University Hospital that occurred in April 2020; at that time the first nationwide outbreak of COVID-19 emerged in Japan. In a 1-month period of the nosocomial infection, over 400 samples from both patients and healthcare workers involved in the crisis were tested. The rapid COVID-19 diagnostics at the center led to appropriate infection control in the university hospital, which contributed to containment of the nosocomial outbreak.

### Pros and cons of diagnostics at academic laboratories

The largest advantage of diagnosing in an academic setting is that the turnaround time is extremely short. It normally takes 2–3 days or more to obtain results when clinical laboratory tests for COVID-19 diagnosis are outsourced. An in-house academic laboratory, on the other hand, can be specialized for a limited number of testing scenarios such as detecting SARS-CoV-2, shifting its human resource, materials, and equipment from research to clinical diagnostics. Documented procedures for testing and transferring patient samples can be simplified because both hospital and laboratory belong to the same organization. These merits enable the hospital to get the result back from the laboratory within a few hours to a day. For example, the turnaround time is 0.36 days on average (as of July 12, 2020) at the PCR center of The Jikei University School of Medicine (as described above). In addition, the cost of testing at in-house laboratories can be more affordable, compared with that of outsourcing the test to commercial clinical laboratories. PCR testing in in-house laboratories may also offer shorter turnaround times and affordable, less expensive diagnosis in other countries.

It is obviously beyond the scope of academic laboratories to handle any clinical samples. It may be a concern that staff (researchers and technicians) at the laboratory, newly starting molecular diagnosis, are afraid not familiar with responsibility and accountability required when they participate in clinical diagnosis, possibly causing higher levels of stress to those staff. A fully integrated automated platform system may release these people from such heavy pressure with laborious manual tasks. It is also absolutely essential that each in-house testing assay must be validated for reagents, machines, and equipment in addition to its procedure. In comparison to typical sample-to-answer assays, a manual assay increases the risk of human error. Given that there are a variety of limitations regarding the specificity and sensitivity of PCR testing, a negative result may not be able to preclude SARS-CoV-2 infection.

## Conclusion and perspectives

The potential advantages of implementing a clinical diagnostic pipeline of COVID-19 in research laboratories in academic settings have been discussed, providing a blueprint to support others in establishing similar structures. Practical examples in a university may be applicable for other smaller institutions such as community hospitals lacking extensive clinical laboratories to run molecular diagnosis with automated equipment. Although the advantages of implementing a clinical diagnostic platform in academic settings are clear, there are still challenges that need to be solved. First, these academic pipelines require clinical method validation and verification, cooperating with clinical diagnostic laboratories. Second, the choice of diagnostic measures should be adapted to local resource and staff expertise within a research laboratory. Third, the academic settings should consider how to ensure medium- to long-term sustainability of the testing platform. In summary, the clinical development and implementation of diagnostic laboratories in academic institutions may offer valuable measures when the world faces another pandemic of emerging and re-emerging infectious disease in the future.

## Data Availability

Not applicable
